# Accuracy of echocardiographic area-length method in chronic myocardial infarction: comparison with cardiac CT in pigs

**DOI:** 10.1186/s12947-016-0093-0

**Published:** 2017-01-09

**Authors:** Haitham Ballo, Miikka Tarkia, Matti Haavisto, Christoffer Stark, Marjatta Strandberg, Tommi Vähäsilta, Virva Saunavaara, Tuula Tolvanen, Mika Teräs, Ville-Veikko Hynninen, Timo Savunen, Anne Roivainen, Juhani Knuuti, Antti Saraste

**Affiliations:** 1 Turku PET Centre, University of Turku and Turku University Hospital, Kiinamyllynkatu 4-8, Turku, 20520 Finland; 2 Heart Center, Turku University Hospital and University of Turku, Turku, Finland; 3 Research Centre of Applied and Preventive Cardiovascular Medicine, University of Turku, Turku, Finland; 4 Department of Anesthesiology, Intensive Care, Emergency Care and Pain Medicine, Turku University Hospital, Turku, Finland; 5 Turku Center for Disease Modeling, University of Turku, Turku, Finland; 6 Institute of Clinical Medicine, University of Turku, Turku, Finland

**Keywords:** Ejection fraction, Transthoracic echocardiography, Cardiac CT

## Abstract

**Background:**

We evaluated echocardiographic area-length methods to measure left ventricle (LV) volumes and ejection fraction (EF) in parasternal short axis views in comparison with cardiac computed tomography (CT) in pigs with chronic myocardial infarction (MI).

**Methods:**

Male farm pigs with surgical occlusion of the left anterior descending coronary artery (*n* = 9) or sham operation (*n* = 5) had transthoracic echocardiography and cardiac-CT 3 months after surgery. We measured length of the LV in parasternal long axis view, and both systolic and diastolic LV areas in parasternal short axis views at the level of mitral valve, papillary muscles and apex. Volumes and EF of the LV were calculated using Simpson’s method of discs (tri-plane area) or Cylinder-hemiellipsoid method (single plane area).

**Results:**

The pigs with coronary occlusion had anterior MI scars and reduced EF (average EF 42 ± 10%) by CT. Measurements of LV volumes and EF were reproducible by echocardiography. Compared with CT, end-diastolic volume (EDV) measured by echocardiography showed good correlation and agreement using either Simpson’s method (*r* = 0.90; mean difference −2, 95% CI −47 to 43 mL) or Cylinder-hemiellipsoid method (*r* = 0.94; mean difference 3, 95% CI −44 to 49 mL). Furthermore, End-systolic volume (ESV) measured by echocardiography showed also good correlation and agreement using either Simpson’s method (*r* = 0.94; mean difference 12 ml, 95% CI: −16 to 40) or Cylinder-hemiellipsoid method (*r* = 0.97; mean difference:13 ml, 95% CI: −8 to 33). EF was underestimated using either Simpson’s method (*r* = 0.78; mean difference −6, 95% CI −11 to 1%) or Cylinder-hemiellipsoid method (*r* = 0.74; mean difference −4, 95% CI–10 to 2%).

**Conclusion:**

Our results indicate that measurement of LV volumes may be accurate, but EF is underestimated using either three or single parasternal short axis planes by echocardiography in a large animal model of chronic MI.

## Background

The biplane method of disks (modified Simpson’s rule) is the currently recommended 2D method to assess left ventricle (LV) volumes and ejection fraction (EF) [1]. Accordingly, LV volumes should be measured from the apical four- and two chamber views. When apical views are not technically feasible or poor apical endocardial definition precludes accurate tracing, an alternative method to calculate LV volumes is the area-length method, in which the LV is assumed to be hemiellipsoid or bullet shaped [1]. The LV cross-sectional area is computed by planimetry in the parasternal short-axis view or views and the length of the ventricle measured from the midpoint of the mitral valve annular plane to the apex. Variations of the method include the use of different mathematical models and LV cross-sectional area in the mid-ventricle only or mid-ventricle, basal and apical levels [2–6].

Area-length methods have been validated ex vivo after formalin fixation or in vivo with x-ray cineangiography or radionuclide techniques in normal hearts and in various pathological conditions [3–8]. However, there is limited data validating the use of area-length method for measurement of LV volumes and EF in the presence of regional wall motion abnormalities caused by chronic myocardial infarction (MI) [2]. Furthermore, the quality of 2D images has improved over time. We hypothesized that area-length method would enable accurate LV volume quantification in parasternal short axis images and that computation of LV cross-sectional area at three levels (modified Simpson’s method) of the LV would be preferable to one level only (Cylinder-hemiellipsoid method) in the presence of MI scar.

The purpose of this study was to validate echocardiographic area-length methods to quantify LV volumes and EF in parasternal short axis and long axis images using dynamic cardiac computed tomography (CT) as a reference in the presence of regional dysfunction caused by chronic MI in a pig model. Furthermore, we compared Simpson’s method of discs and Cylinder-hemiellipsoid methods, which are based on LV area measurements at the level of mitral valve, papillary muscles and apex or at the level of papillary muscles only, respectively.

## Methods

### Experimental animals and general study protocol

Male Finnish Landrace pigs [age 3 months, weight 28 ± 4 kg (range 19–43 kg)] had either a sham operation (control group) or a concurrent 2-step occlusion of the left anterior descending (LAD) coronary artery with distal ligation for the preconditioning of the heart and subsequent implantation of a proximal ameroid constrictor (chronic MI group) as described recently. The ameroid constrictor will occlude resulting in large MI [9]. After a 3-month follow-up, echocardiography, CT and positron emission tomography (PET) were performed in the same imaging session.

All pigs were housed and fed in individual pens under a 12-h light/12-h dark cycle. Animals were fed with normal farm pig diet (Pekoni 90, Hankkija-Maatalous Oy, Hyvinkää, Finland). Water was provided ad libitum. Animal health was monitored on a daily basis. All animal experiments were made according to European Community Guidelines for the use of experimental animals and approved by Finnish National Animal Experiment Board.

There were 21 pigs in the chronic MI group that survived the 3-month follow-up until the imaging studies. Detailed procedural and long-term survival in this model has been reported previously [9]. Six pigs were excluded due to inability to obtain echo images because of logistic reasons or inadequate imaging windows related to the surgery scar. Another 6 were excluded due to inability to perform cardiac CT due to logistic reasons. Ten animals had a sham operation. One sham-operated animal was sacrificed due to oesophageal obstruction. Three were excluded due to missing or incomplete echocardiographic images. One was excluded due to inability to perform cardiac CT due to logistic reasons. Thus, the final study group consisted of 9 pigs with MI and 5 sham-operated controls.

### Anaesthesia and hemodynamic monitoring

Prior to the surgical operation or imaging studies, animals were anaesthetized with intramuscular (i.m.) administration of midazolam 1 mg/kg (Midazolam Hameln, Hameln Pharmaceuticals GmbH, Hameln, Germany) and xylazine 4 mg/kg (Rompun vet, Bayer Animal Health GmbH, Leverkusen, Germany) and connected to a respirator and ventilated mechanically (tidal volume 8–10 mL/kg, frequency 14–18 min^−1^, Dräger Oxylog 3000, Drägerwerk AG, Lübeck, Germany). An ear vein was cannulated using a 22G venous catheter and anesthesia was maintained with intravenous (i.v.) infusion of propofol 10–50 mg/kg/h (Propofol Lipuro, B. Braun Melsungen AG, Melsungen, Germany) combined with fentanyl 4–8 μg/kg/h i.v. (Fentanyl-Hameln, Hameln Pharmaceuticals GmbH, Hameln, Germany). Femoral artery was cannulated for hemodynamic monitoring during the imaging studies. Diastolic, systolic and mean arterial pressure and heart rate were recorded using a pressure transducer (TruWave, Edwards Lifesciences Corp., Irvine, CA, USA) connected to an anesthesia monitor (Datex Ohmeda S5, GE Healthcare Finland Oy, Helsinki, Finland).

### Surgical operation and medication

Animals were operated on as previously described [9]. A short left anterior thoracotomy was performed to allow a direct view of the LAD. The pericardium was opened and tented and a complete ligation of the distal LAD was made immediately after the second diagonal branch using a 5-0 monofilament polypropylene suture (Prolene, Ethicon, and Norderstedt, Germany). Approximately 15 min later, the proximal LAD was prepared free and an ameroid constrictor (2.50 mm or 2.75 mm, model MRI-2.50-TI and MRI-2.75-TI; Research Instruments SW, Escondido, CA, USA) was placed around the LAD. Ameroid size was selected on the basis of the diameter of the LAD. In the control group, a sham operation including thoracotomy and pericardial dissection without the LAD occlusion or implantation of the ameroid was performed.

For analgesia, fentanyl 4–8 μg/kg i.v. was administered intraoperatively and fentanyl 2–4 μg/kg/h (Matrifen transdermal patch, Takeda Pharma A/S, Roskilde, Denmark) postoperatively for 3–7 days. Bupivacain 25 mg i.m. (Bicain, Orion Pharma, and Espoo, Finland) was administered locally to anesthetize the thoracotomy wound at the end of the operation. A single dose of cefuroxime 30 mg/kg i.v. (Cefuroxime, Orion Pharma, and Espoo, Finland) was administered preoperatively for antibiotic prophylaxis. In order to prevent ventricular arrhythmias, amiodarone (Cordarone, Sanofi-Synthelabo Ltd, Newcastle upon Tyne, UK) was administered 8 mg/kg perorally (p.o.) daily for 1 week before and for 2 weeks after the surgery. Amiodarone 6 mg/kg i.v, metoprolol 0.2 mg/kg i.v. (Seloken, Genexi, Fontenaysous Bois, France) and magnesium sulphate (MgSO_4_) 25 mg/kg i.v. (Addex-magnesiumsulfaatti, Fresenius Kabi AB, Uppsala, Sweden) were administered intraoperatively. Clopidogrel 3 mg/kg p.o. (Plavix, Sanofi Winthrop Industrie S.A., Ambarès et Lagrave, France) was administered daily 1 day before and daily for 2 weeks after the surgery to prevent premature thrombosis of the LAD.

### Transthoracic echocardiography

Echocardiographic studies were performed by a portable Vivid Q device and MS5 transducer (GE, Hjorten, Norway). The anesthesized animals were studied in supine position. Left or right parasternal views were used to visualize the LV. 2D parasternal long axis view including the apex and short-axis views obtained at the level of the mitral valve (basal LV level), papillary muscles (papillary level), and apex (apical level). All images were stored in DICOM format, and analysed off-line using Echo PAC PC 113 software (GE, Hjorten, Norway).

### Echocardiography image analysis

Area of the LV cavity was measured planimetrically by manually tracing the endocardial borders in short axis views at the level of mitral valve, papillary muscles and apex. Papillary muscles and trabeculations were included within the cavity. End-diastole was identified at the beginning of the QRS complex of the simultaneously recorded electrocardiography (ECG) and the systolic frame was selected as the one with smallest LV cavity. LV length was measured in parasternal long axis view from the apex to the level of the mitral valve annulus. Each measurement was repeated 2 times and mean of them recorded.

Reproducibility of the measurements was tested by repeated analysis of images of 5 pigs by the same or two independent observers and coefficient of variation (CV) was calculated.

The end-diastolic volume (EDV), end-systolic volume (ESV) and EF were calculated using two different methods:

- The modified Simpson’s method [3–6]: The volume of the two basal thirds of LV is determined using the Simpson’s rule, but volume of apex is estimated separately as the volume of an ellipsoid volume segment using formula:
  $$ \mathrm{Volume}=\left({\mathrm{A}}_1+{\mathrm{A}}_2\right)\left(\mathrm{L}/3\right)+\left({\mathrm{A}}_3/2\right)\left(\mathrm{L}/3\right)+\left(\uppi /6\right){\left(\mathrm{L}/3\right)}^3. $$
  Where A _1_, A_2_, and A_3_ are LV areas at the level of mitral valve, papillary muscles and apex; and L is the maximum length of ventricle
- Cylinder-hemiellipsoid [3–5]: Is a combined figure model in which the LV is divided into cylinder and hemiellipsoid. The volume is calculated from the formula.
  $$ \mathrm{Volume}=5/6\mathrm{A}\mathrm{L}. $$
  Where A is LV cavity area at the level of papillary muscles and L length of the LV.

The LV EF was then calculated according to formula:

$$ \frac{\mathrm{End}\hbox{-} \mathrm{diastolic}\ \mathrm{volume}-\mathrm{End}\hbox{-} \mathrm{systolic}\ \mathrm{volume}}{\mathrm{End}\hbox{-} \mathrm{diastolic}\ \mathrm{volume}}\times 100 $$

### Dynamic cardiac CT

End-diastolic and end-systolic volumes (EDV, ESV) and ejection fraction (EF) were evaluated by helical computed tomography angiography (CTA, GE Discovery VCT, General Electric Medical Systems, Wankesha, WI, USA) with iodinated contrast agent (Omnipaque 350 mg I/mL, Amersham Health AS, Nydalen, Oslo, Norway). Contrast agent (100 mL) was administered at 4 mL/s via the ear vein and flushed with 100 mL of physiological saline. CTA scanning was performed during breath-hold and started immediately when contrast agent appeared into the LV. A 3-lead ECG was used for cardiac triggering and CTA images were reconstructed with retrospective gating at 0–90% at 10% interval relative to the cardiac cycle. Left ventricular EDV and ESV were calculated by tracing the endocardial borders with semi-automated analysis software (CardIQ Function Xpress) and ADW 4.5 work station (GE Medical Systems, Milwaukee, WI, USA).

### [^11^C]acetate PET

Size of the MI was defined by PET perfusion imaging with [^11^C]acetate as described [9]. [^11^C]acetate [782 ± 65 MBq (range 689–874 MBq)] was injected i.v. via the ear vein as a slow bolus. The acquisition frames were as follows: 10 × 10 s, 1 × 60 s, 5 × 100 s, 5 × 120 s, 5 × 240 s (total duration 41 min). The acquired PET data was reconstructed with an iterative VUE Point algorithm. There were 2 iterations and 24 subsets in reconstruction. The whole transaxial field of view (70 cm) was reconstructed in 128 × 128 matrix yielding pixel size of 5.47 mm × 5.47 mm. The measurements were corrected for scatter, random counts and dead time. The device produces 47 axial planes with a slice thickness of 3.27 mm. Images were analysed using cardiac image analysis Carimas 2 software (Turku PET Centre, Turku, Finland; http://www.turkupetcentre.fi/carimas), polar maps of myocardial blood flow were generated, and MI was defined as the area of the LV (%) with resting perfusion <60% of the maximum.

### Tissue samples

Immediately after the imaging studies, the animals were sacrificed by i.v. injection of potassium chloride. Heart was excised and sliced horizontally to four slices from base to apex.

In order to confirm the presence of MI, samples were incubated for 15 min in 1% 2,3,5-triphenyltetrazolium chloride (TTC) (Sigma-Aldrich, Saint Louis, MO, USA) diluted in phosphate-buffered saline (pH 7.4) at 37 °C. Stained myocardial samples were photographed from both sides.

### Statistical analysis

All data were expressed as mean ± SD. Linear regression analysis and Pearson’s correlation were used to compare CT and two-dimensional echocardiography. The degree of agreement was analyzed by using Bland-Altman method. Student’s *t*-test for non-paired data was used to compare means of two groups. *p* value > 0.05 was considered as significant.

## Results

### Basic characteristics of animals

The final study group consisted of 9 pigs with coronary ligation and 5 sham-operated controls. Mean weight was 104 ± 16 kg (range 84–130 kg). The hemodynamic parameters measured at the time of imaging are shown in Table 1.

**Table 1:** Hemodynamic characteristics of pigs with myocardial infarction (MI) and controls

Cardiovascular index	Control (*n* = 5)	MI (*n* = 9)	All (*n* = 14)	*p*
Heart rate (bpm)	110 ± 18	86 ± 18	95 ± 21	0.16
Systolic blood pressure (mmHg)	142 ± 18	120 ± 18	128 ± 20	0.19
Diastolic blood pressure (mmHg)	96 ± 6	78 ± 15	84 ± 15	0.12

None of the sham-operated pigs had MI, whereas an area of MI was detected in the apical and/or mid-ventricular slices of the anterior septum and anterior wall in 8 animals with an ameroid constrictor implanted based on TTC staining of myocardial slices. In these pigs, the MI size varied from 3 to 57% of the LV and the average size was 23 ± 19% as shown by PET perfusion imaging.

### Measurement of LV volumes and EF

**Fig. 1:**
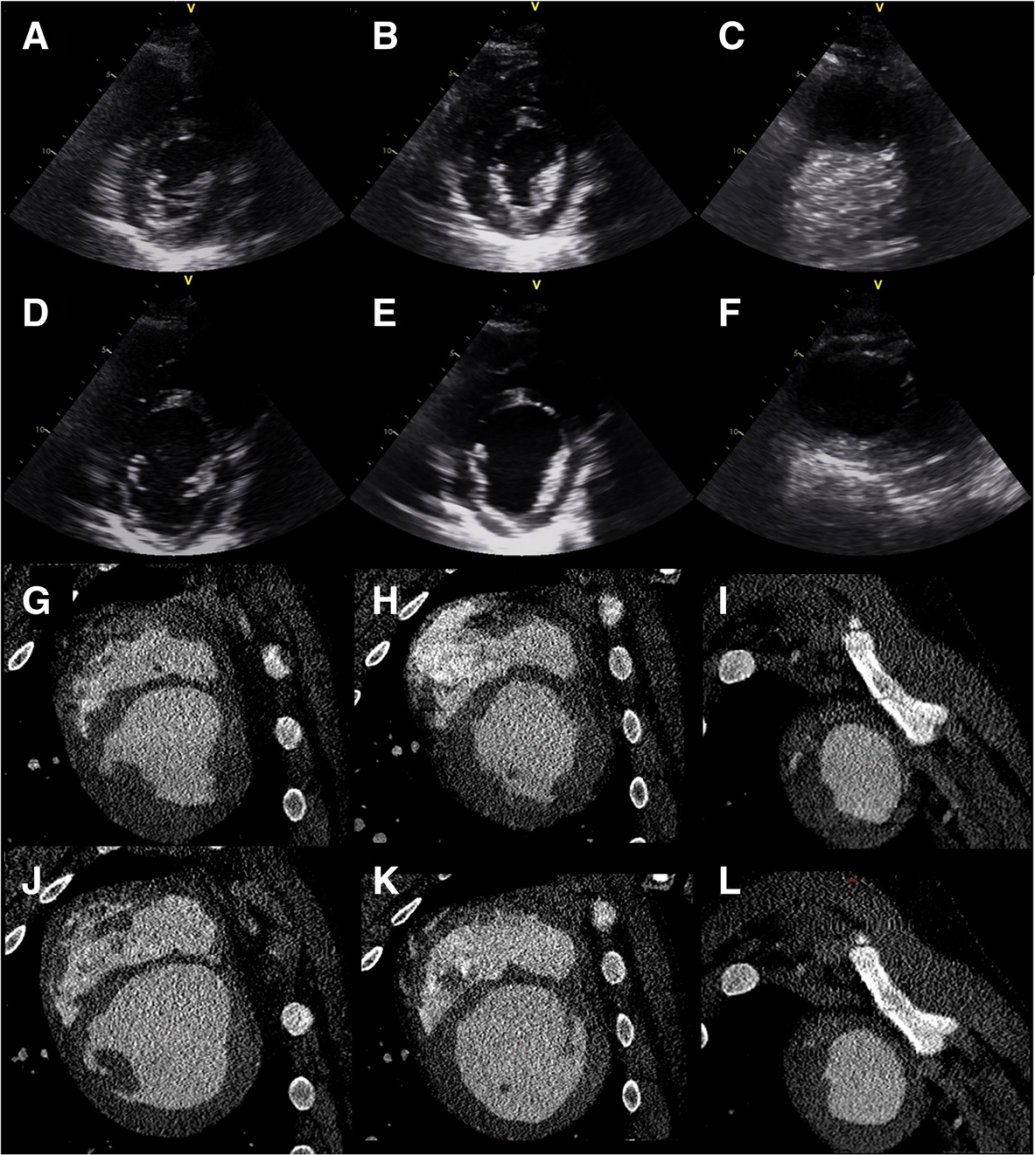
Echocardiographic parasternal short-axis views of the left ventricle (LV) at the level of mitral valve (**a** and **d**), papillary muscles (**b** and **e**) and apex (**c** and **f**) at systole (**a**, **b** and **c**) and end-diastole (**d**, **e** and **f**). Short axis cardiac CT views of the LV of the same pig at the level of mitral valve (**g** and **j**), papillary muscles (**h** and **k**) and apex (**i** and **l**) at systole (**g**, **h** and **i**) and end-diastole (**j**, **k** and **l**). The pig had myocardial infarction involving of45% of the LV

Representative echocardiographic and CT images used for delineation of the LV cavity are shown in Fig. 1. LV diastolic volume, systolic volume and EF by cardiac CT and echocardiography using either Simpson’s method or Cylinder-hemiellipsoid method are shown in Table 2. In pigs with MI, average LV volumes or EF measured by echocardiography did not differ significantly from those measured by CT (Table 2). Although LV diastolic volumes were similar in pigs with MI and controls, systolic volumes were larger and EF was lower in pigs with MI than controls.

**Table 2:** Left ventricle volumes at diastole (EDV) and systole (ESV) and ejection fraction (EF) measured by cardiac CT or echocardiography and either Simpson’s, or Cylinder-hemiellipsoid methods in pigs with chronic myocardial infarction (MI) and controls

	Control (*n* = 5)	MI (*n* = 9)	All (*n* = 14)	*p*
EDV (mL)				
Cardiac CT	140 ± 19	227 ± 96	196 ± 88	0.07
Modified Simpson’s	124 ± 18	225 ± 126 ^a^	189 ± 111	0.1
Cylinder hemiellipsoid	112 ± 22	230 ± 140 ^b^	188 ± 125	0.09
ESV (mL)				
Cardiac CT	49 ± 2	137 ± 79	106 ± 76	0.03
Modified Simpson’s	59 ± 14	149 ± 98 ^c^	117 ± 89	0.07
Cylinder hemiellipsoid	47 ± 14	150 ± 94 ^d^	113 ± 90	0.03
EF (%)				
Cardiac CT	64 ± 4	42 ± 10	50 ± 14	<0.001
Modified Simpson’s	53 ± 5	37 ± 8 ^e^	42 ± 11	0.0015
Cylinder hemiellipsoid	59 ± 7	38 ± 6 ^f^	46 ± 12	<0.001

^a^ *P* = 0.86 vs. Cardiac CT, ^b^ *P* = 0.85 vs. Cardiac CT, ^c^ *P* = 0.72 vs. Cardiac CT, ^d^ *P* = 0.82 vs. Cardiac CT, ^e^ *P* = 0.1 vs. Cardiac CT, ^f^ *P* = 0.3 vs. Cardiac CT

Reproducibility of repeated measurements by the same or two observers are shown in Table 3. CV was always lower than 11% indicating good reproducibility.

**Table 3:** Coefficients of variation (CV) between repeated measurements by the same (Intra-observer) and two (Inter-observer) observers in 5 pigs

Echocardiographic index	Intra-observer	Inter-observer
	CV (%)	CV (%)
Modified Simpson’s		
Ejection fraction	8.2	10.4
End-diastolic volume	2.5	6.4
End-systolic volume	4.7	10.9
Cylinder hemiellipsoid		
Ejection fraction	9.3	10.4
End-diastolic volume	1.9	10.3
End-systolic volume	2.5	6.4

### Agreement between echocardiography and cardiac CT

**Fig. 2:**
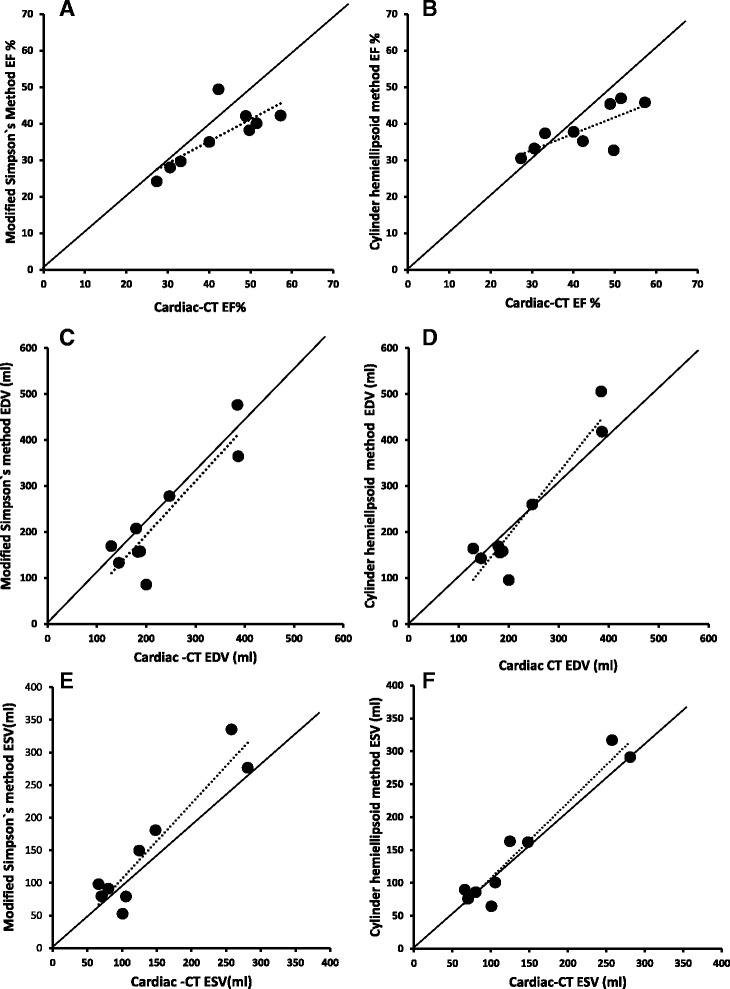
Correlations between ejection fraction (EF, **a** and **b**), end-diastolic volume (EDV, **c** and **d**) and end-systolic volume (ESV, **e** and **f**) measured by cardiac computed tomography (Cardiac-CT) and echocardiography using either the modified Simpson’s method (**a**, **c**, **e**) or cylinder hemiellipsoid method (**b**, **d**, **f**) in pigs with MI

The correlations between LV volumes and EF measured by echocardiography and dynamic cardiac CT are shown in Fig. 2. There were good correlations between LV diastolic volumes measured with CT and either the Simpson’s (*r* = 0.90, *p* = 0. 001) or Cylinder-hemiellipsoid (*r* = 0.94, *p* = 0. 0002) methods. good correlations were also found between LV systolic volumes measured with CT and the Simpson’s (*r* = 0.94, *p* = 0. 0003) or Cylinder-hemiellipsoid (*r* = 0.97, *p* < 0.0001) methods. There was a relatively good correlation between EF measured by CT and Simpson’s method (*r* = 0.78, *p* = 0. 01) or Cylinder-hemiellipsoid method (*r* = 0.74, *p* = 0. 02).

**Fig. 3:**
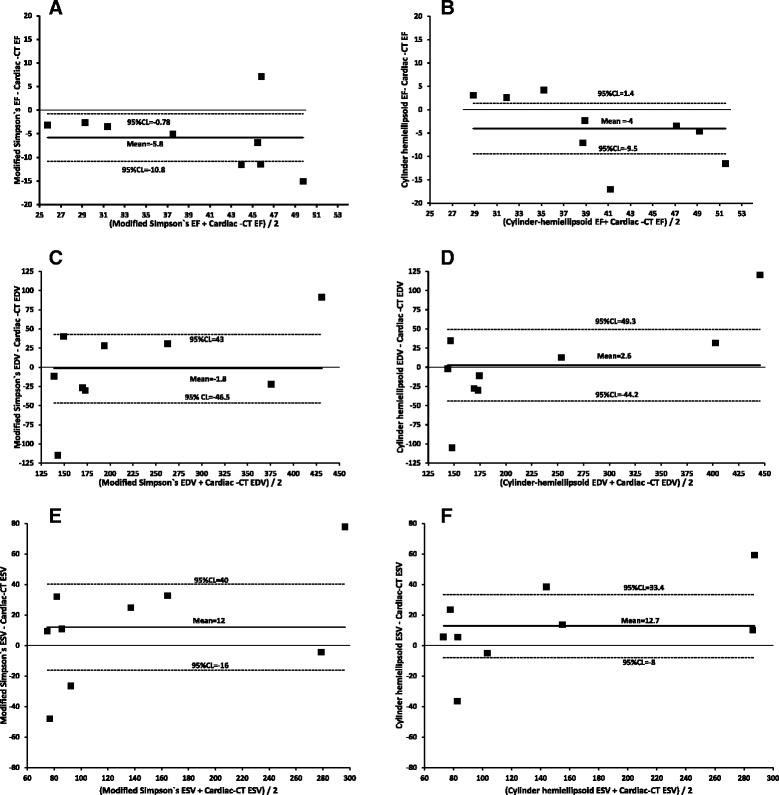
Bland-Altman analysis for agreement between ejection fraction (EF, **a** and **b**), end-diastolic volume (EDV, **c** and **d**) and end-systolic volume (ESV, **e** and **f**) measured by cardiac computed tomography (Cardiac-CT) and echocardiography using either the modified Simpson’s method (**a**, **c**, **e**) or cylinder hemiellipsoid method (**b**, **d**, **f**) in pigs with MI

Bland-Altman plots between LV volumes and EF measured by CT or echocardiography are shown in Fig. 3. There was a good agreement between LV diastolic volumes measured by CT and either Simpson’s method (mean difference: −2 ml, 95% CI: −47 to 43) or Cylinder-hemiellipsoid method (mean difference: 3 ml, 95% CI: −44 to 49). Compared with CT, LV systolic volumes were overestimated by the Simpson’s method (mean difference: 12 ml, 95% CI: −16 to 40) and by Cylinder-hemiellipsoid method (mean difference: 13 ml, 95% CI: −8 to 33). Compared with CT, both Simpson’s method and Cylinder-hemiellipsoid method underestimated the LV EF (mean difference: −6%, 95% CI: −11% to 1%and −4%, 95% CI: −10% to 2%, respectively).

## Discussion

These results suggest that area-length methods obtained using parasternal short axis projections are valid alternatives for the assessment of LV volume, although EF was appears to be underestimated in the presence of chronic MI when apical views are not technically feasible.

We found that LV volumes and can be measured relatively accurately, but EF is underestimated using echocardiography and area-length methods in the presence of regional wall motion abnormality due to chronic MI. Comparable estimates can be obtained with hemiellipsoid method and modified Simpson’s methods when CT was used as a reference. We used a novel pig model of chronic MI. In this model, ameroid constrictor gradually occludes the LAD resulting in large MI. Two-step occlusion improves survival of animals and enables long-term follow-up studies. The technique resulted in variable MI size and none of the pigs showed signs of overt heart failure. Although systemic hemodynamic were variable, there was a tendency towards lower heart rate and blood pressure in pigs with MI that may be related to enhanced cardioinhibitory response to the anaesthetic agents in the presence of cardiac dysfunction. As in many other animal models, apical echocardiographic windows are not possible to obtain in pig due to chest anatomy that necessitates the use of parasternal views only. Therefore, our findings have implications for translational studies using pig models of MI and ischemic heart failure.

Area-length methods have been previously validated in ex vivo formalin fixed hearts and in patients using X-ray cineangiography or radionuclide ventriculography as a gold standard [3–8]. The patient series included total number of approximately 50 patients with different pathologies, including as ischemic heart disease, cardiomyopathy, valvular heart disease, constrictive pericarditis or hypertrophy [6–8]. Regional wall motion abnormalities were reported to be present in many patients. One experimental study has been performed after acute (1 h) occlusion of the LAD in dog. In these studies, reasonably good correlations were obtained between area-length echocardiography and the reference standard using either Simpsons’s technique or the hemiellipsoid method. In the presence of LAD occlusion, both Simpson’s technique and the hemiellipsoid technique provided good correlations when short axis image from the papillary muscle level including the region with wall motion abnormality was included [8]. Our study adds to the previous studies by systematically comparing area-length method with cardiac CT in the presence of LV dysfunction caused by chronic MI that is a common cause of heart failure. Quantification of LV volume and EF by CT is based on delineation of actual chamber volumes that has been shown to be accurate when compared with magnetic resonance imaging (MRI) [10]. However, cardiac MRI is considered as the gold standard for this purpose [11, 12].

As in previous studies we found that volumes were accurately measured by the area-length method. In this study, we measured the longest length of the LV from the apical long axis view. This is in line with a previous study recommending the use of the apical long-axis view due to low risk of foreshortening of the image, and providing an additional endocardial reference position in the aorta [13]. In pig, it was feasible to visualize the entire length of the LV except a small tip in the apex in some animals from parasternal long axis view.

Simpson’s method and the cylinder hemiellipsoid methods were used in the present study since they have shown good accuracy in previous studies. We also tested cylinder truncated cone and cylinder cone-cone models (data not shown), but their performance in measuring LV volumes was inferior to Simpson’s or cylinder hemiellipsoid methods.

### Study limitations

Although reproducibility of measurements was good, high operative mortality resulted in small sample size that can affect comparisons of small differences between methods [9]. Therefore, the results should be confirmed in a larger series. In general, the accuracy of estimation of left ventricular volumes and EF on 2D echocardiography is limited by geometrical assumptions, standardized image positions with respect to LV long axis, and accurate tracing of LV endocardial boundaries [5, 7, 14]. In addition, measurement of long axis length may be affected, because the cardiac apex not always visualized in the long axis view.

## Conclusions

Our results indicate that measurement of LV volumes may be accurate, but EF is underestimated in parasternal short axis images using transthoracic echocardiography in a large animal model of chronic MI.

## Abbreviations

CT: Computed tomography
CTA: Computed tomography angiography
CV: Coefficient of variation
ECG: Electrocardiography
EDV: End-diastolic volume
EF: Ejection fraction
ESV: End-systolic volume
LAD: Left anterior descending coronary artery
LV: Left ventricle
MI: Myocardial infarction
MRI: Magnetic resonance imaging
PET: Positron emission tomography
TTC: 2,3,5-triphenyltetrazolium chloride
TTE: Transthoracic echocardiogram
